# Japanese Legacy Cohorts: A New Series of Special Articles Has Started

**DOI:** 10.2188/jea.JE20180027

**Published:** 2018-04-05

**Authors:** Kota Katanoda

**Affiliations:** Division of Cancer Statistics Integration, Center for Cancer Control and Information Services, National Cancer Center, Tokyo, Japan

A new series of articles on “Japanese legacy cohorts” has started from this issue of the *Journal of Epidemiology*. The first memorable article of this series is about the “Life Span Study,” the cohort of the Hiroshima and Nagasaki atomic bomb survivors.^1^ This special article by Ozasa et al sends us back to the era more than 70 years ago. This cohort study was started in the 1950s in the midst of postwar chaos and is still following the survivors. Needless to say, it has produced massive scientific evidence that is the basis of current radiation protection guidelines. As noted in the special article, approximately 214,000 people in Hiroshima and Nagasaki died by the end of the year of bombings, and 284,000 people were identified as survivors. The knowledge of radiation protection, from which we benefit today, has been obtained through the sacrifice and dedication of their lives. Science produced the devastating weapons, but it is also science that produced knowledge for radiation protection, for which epidemiology has played an important role.

According to Sir Richard Doll, a giant in epidemiology, the term “cohort study” appeared for the first time in a study by Frost in the 1930s.^2^^,^^3^ In his study, Frost investigated the incidence rates of tuberculosis by age group according to birth years; thus, the concept is rather close to a “birth cohort effect”. Approximately 20 years later, around 1950, a “cohort study” in the current meaning (referred to as prospective cohort studies throughout the paper) was initiated in the United Kingdom by Sir Doll himself called the “British doctors study”.^4^ This study was designed to investigate the relationship between smoking and lung cancer. Following the observation of a clear relationship between smoking and lung cancer in the case-control study performed by Sir Doll himself,^5^ a prospective verification study was planned under the advice of the co-author Sir Austin Bradford Hill.^2^ Similarly, in around 1950, two cohort studies were initiated in the United States. One is the “American Cancer Society Study”,^6^ which targeted cancer, and the other is the “Framingham Study”,^7^ which targeted cardiovascular diseases. Meanwhile, in the United Kingdom, the “1946 Birth birth-cohort study,” studying the cohort of new born babies in 1946, was initiated, which is still continuing.^8^^,^^9^ An article about this study was published in *Nature* in 2011, 65 years after the initiation.^10^

In Japan, too, historic cohort studies were initiated in the 1950s and 1960s. This series of “Japanese legacy cohorts” was designed to provide learning opportunities widely to the readers by revisiting these cohort studies from an historical perspective. In this series, the following four cohort studies will be presented:

1. Life Span Study (in this issue)
2. Hisayama Study
3. Circulatory Risk in Communities Study (CIRCS)
4. Hirayama Cohort (Keikaku Chosa)

These four cohort studies are quite unique worldwide and have produced scientific knowledge that can greatly change not only medical practices but also our life and social system. It is the hope of all *Journal of Epidemiology* editors that this series will provide the readers with great opportunities to learn about the history of epidemiology and develop future ideas based on past experiences.
